# Pharmacokinetics and bioequivalence assessment of two prucalopride formulations in healthy Chinese women: a randomized, open-label, two-period, two-sequence, self-crossover study

**DOI:** 10.3389/fphar.2025.1562692

**Published:** 2025-04-23

**Authors:** Xiangxin Huang, Ying Wang, Bei Li, Xiaoqun Shen, Xuexia Tao, Wenwen Zheng, Qi Luo, Lei Xiong, Lin Wang, Shufan Cai

**Affiliations:** Department of Clinic Trial, Affiliated Hangzhou First People’s Hospital, School of Medicine, Westlake University, Hangzhou, China

**Keywords:** bioequivalence, prucalopride succinate, pharmacokinetics, healthy Chinese women, crossover clinical trial

## Abstract

**Objective:**

This study aimed to evaluate the pharmacokinetic (PK) bioequivalence of generic and branded prucalopride formulations.

**Methods:**

Twenty-four healthy female subjects were enrolled in both fasted and fed trials, with each subject receiving either the test (generic) or reference (branded) formulation after an overnight fast. Blood samples were collected up to 72 h post-administration. Plasma concentrations of prucalopride were quantified using ultra-performance liquid chromatography–tandem mass spectrometry (UPLC-MS), and the corresponding PK parameters were subsequently calculated. Clinical safety data were monitored throughout the trial period.

**Results:**

All 24 subjects completed both the fasted and fed trials. No significant differences were found in the PK data between the test and reference formulations for either the fasted or fed states. The Wilcoxon signed-rank test of T_max_ revealed no significant differences between the two formulations in both the fasted (*P* = 0.319) and fed (*P* = 0.973) states. The 90% confidence intervals (CIs) for the bioequivalence parameters fell within the 80%–125% range, which meets the standard bioequivalence acceptance criteria. Additionally, there were no significant differences in the incidence of adverse events (AEs) between the generic and branded formulations, and no serious AEs were reported throughout the trial period.

**Conclusion:**

The generic and branded prucalopride tablets were bioequivalent in terms of PK parameters and demonstrated no clinically relevant differences in safety outcomes.

**Clinical Trial Registration:**

http://www.chinadrugtrials.org.cn/clinicaltrials.prosearch.dhtml, identifier CTR20232669.

## Highlights

• This study focused on healthy Chinese women to assess the pharmacokinetics and safety of the generic prucalopride formulation.
• Both the test and reference formulations of prucalopride tablets were found to be bioequivalent and well tolerated in the healthy Chinese female subjects.

## 1 Introduction

Constipation is one of the most common digestive complaints in the general population (Wald et al., 2007; Long et al., 2017; Zhao et al., 2011). It is characterized by symptoms such as hard stools, excessive straining, infrequent bowel movements (fewer than three per week), a sensation of incomplete evacuation, and/or the use of digital maneuvers to facilitate defecation. Chronic constipation is diagnosed when these symptoms persist for more than 3 months. Notably, contemporary dietary patterns, which increasingly favor high-sugar and low-fiber foods, may impair gastrointestinal motility and contribute to the rising prevalence of constipation. Studies have reported significant variations in the prevalence of chronic constipation, influenced by factors such as geographical location, population demographics, sampling methodologies, and differing diagnostic criteria. Currently, the average global prevalence of chronic constipation among adults is approximately 16% (Wald et al., 2007). In China, the prevalence ranges from 4.0% to 10.0% (Long et al., 2017; Zhao et al., 2011), while among the elderly population, this proportion approaches 23.0% (Chu et al., 2014). A meta-analysis of 26 studies demonstrated that women have a significantly higher prevalence of chronic constipation than men (17.4% versus 9.2%, odds ratio 2.2, 95% CI 1.87–2.62) (Suares and Ford, 2011). A multicenter global survey revealed that the quality of life in patients with chronic constipation is significantly lower than in those without the condition (Wald et al., 2007). Chronic constipation management can also lead to substantial economic burdens for patients, primarily due to laxative overuse and recurrent healthcare utilization (M et al., 2018).

The mean medications used to treat chronic constipation mainly include laxatives, stimulant laxatives, surfactants, osmotic agents, guanylate cyclase-C receptor agonists, and 5-hydroxytryptamine receptor 4 (5-HT4) prokinetic agents (Cj and Ac, 2018). Prucalopride, a selective 5-HT4 receptor agonist, enhances gastrointestinal motility by stimulating propulsive contractions throughout the gastrointestinal tract. It is recommended for patients with chronic idiopathic constipation who are refractory to first-line osmotic or bulk-forming laxatives. In randomized controlled trials, prucalopride (1–4 mg once daily) demonstrated superiority over placebo and exhibited a favorable safety profile in elderly patients (aged ≥65 years) (Yiannakou et al., 2015; Camilleri et al., 2010).

Prucalopride was approved for marketing through centralized review by the European Medicines Agency on 14 October 2009, under the trade name Resolor^®^. It was also approved by the National Medical Products Administration (NMPA) in China on 31 December 2012. Despite being marketed in China, Resolor^®^ remains inaccessible to many patients with chronic constipation, particularly in underserved regions. Heze Pharmaceutical Technology Co., Ltd. (Zhejiang Province, China) developed a generic formulation of prucalopride using the direct compression technology. This formulation contains 1 mg prucalopride succinate and has an excipient composition similar to that of Resolor^®^ (Agency, 2009). This clinical trial was a premarketing study aiming to evaluate the PK, clinical safety, and bioequivalence of this new formulation in comparison to Resolor^®^ in healthy Chinese female subjects under both fasted and fed states.

## 2 Methods and materials

### 2.1 Subjects

Healthy Chinese women aged 18–65 years, with body weight ≥45 kg and body mass index (BMI) between 19.0 and 26.0 kg/m^2^, were included in this study.

Subjects with any of the following were excluded: having a history of allergy to prucalopride or any of its active ingredients or excipients; difficulty in swallowing or having lactose intolerance (i.e., a history of diarrhea after consuming milk); having any chronic or severe diseases affecting the endocrine, urinary, digestive, hematologic, lymphatic, respiratory, cardiovascular, nervous, psychiatric, or musculoskeletal systems; excessive consumption of tea, coffee, or caffeinated beverages (more than eight cups per day, with 1 cup = 250 mL) in the 3 months prior to screening; smoking more than five cigarettes per day in the 3 months prior to screening or showing unwillingness to stop using tobacco products from screening to the end of the study; having had recent vaccinations; having participated in other drug or medical device clinical trials; having previous surgery or plans to undergo surgery during the study period; and consumption of alcohol exceeding 14 units per week in the 6 months prior to screening (1 unit of alcohol = 360 mL of beer, 45 mL of 40% alcohol, or 150 mL of wine). Additionally, subjects were excluded if they had used any medications in the 14 days before screening; taken any medications that interact with or alter the liver enzyme activity related to prucalopride in the 30 days before screening; had an inability to tolerate venipuncture; had a history of needle or blood phobia; or failed venous assessment. Individuals with clinically significant abnormalities detected during physical examination, vital sign assessment, clinical laboratory test (including complete blood count, urinalysis, blood biochemistry, coagulation function, hepatitis and HIV testing, syphilis specificity antibody, and human chorionic gonadotropin levels), or a 12-lead electrocardiogram were also excluded. Female subjects who were pregnant, breastfeeding, or likely to become pregnant were excluded.

### 2.2 Study design and treatment

**FIGURE 1:**
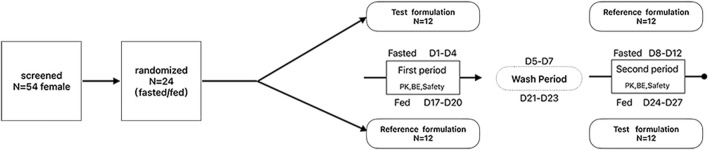
The study flow chart under the fasted and fed states.

This study was designed as a randomized, open-label, single-dose, two-period, two-sequence, and two-treatment crossover bioequivalence study. A total of 24 female subjects were enrolled in both the fasted and fed state trials. The subjects were randomly assigned to either the test–reference (T-R) or reference–test (R-T) drug groups. Each subject fasted overnight for 10 h before each dosing period. In the fed state, a high-fat meal was consumed within 30 min prior to dosing. Each subject received one tablet of the test formulation or the reference formulation (Resolor^®^) with 240 mL of warm water and remained seated throughout the entire administration process under supervision. Water intake was prohibited for 1 h before and after dosing, and lunch was provided 4 h after dosing. The same group of subjects participated in both the fed and fasted state trials, with a 7-day washout period between the two. The specific procedure is outlined in Figure 1.

### 2.3 Blood sample collection

For both the fasted and fed state trials, blood samples were collected at 0 h (30 min before administration) and at the following time points post-administration: 0.33, 0.67, 1, 1.5, 2, 2.5, 3, 3.5, 4, 4.5, 5, 6, 8, 12, 24, 48, and 72 h. Four milliliters of blood was collected into vacuum blood collection tubes containing EDTA as the anticoagulant. The plasma samples were then centrifuged at 1,700 *g* for 10 min at 2°C–8°C using a centrifuge (#ST16R, Thermo Fisher Scientific, United States), aliquoted into detection and backup tubes, and stored at −80°C.

### 2.4 Analytical methodology

Fifty microliters of the plasma sample along with the internal standard (IS) prucalopride-^13^C,d_3_ (#HY-14151S, MedChemExpress, China) was processed via protein precipitation using acetonitrile (#1.00029, Merck KGaA, Germany) at a ratio of 1:5 (v/v). After vortex mixing and centrifugation (10,000*g*, 10 min, 4°C), 200 μL of the supernatant was transferred to a clean tube and evaporated under a controlled nitrogen stream at 35°C. The residue was reconstituted in 150 μL of acetonitrile/water (2:1, v/v), followed by centrifugation (4,000*g*, 5 min, 4°C). A 5-μL aliquot of each sample was subsequently analyzed using a UPLC-MS system (#LCMS-8045, Shimadzu Corporation, Japan).

Quantitative mass spectrometry analysis was performed using an electrospray ionization source in the negative ion mode with multiple reaction monitoring for the transitions *m/z* 368.2→207.1 for prucalopride and *m/z* 371.2→210.1 for IS. Chromatographic separation was achieved on a Shim-pack GIST HP C18 chromatographic column (4.6 mm × 150 mm, 3 μm particle size; #227-30041-05, Shimadzu Corporation, Japan) with a mobile phase consisting of 0.1% formic acid (#27001, Merck KGaA, Germany) in water (A) and acetonitrile (B). The gradient elution program was as follows: 5% B (0–1 min), 5%–95% B (1–4 min), and 95% B (4–5.5 min). The flow rate was set at 0.4 mL/min.

### 2.5 Clinical safety monitoring

Safety monitoring was based on vital signs, physical examinations, laboratory analyses, and 12-lead ECG findings. Abnormal findings observed post-dose and deemed clinically significant according to protocol-defined criteria were recorded as adverse events (AEs) by the investigators. Detailed records of the occurrence time, severity, duration, interventions taken, and outcomes of AEs were maintained throughout the trial. The severity of AEs was assessed according to the Common Terminology Criteria for Adverse Events, version 5.0.

### 2.6 Datasets and statistical analyses

This study employed various datasets for statistical analysis. The full analysis set (FAS) comprised all randomized subjects and was used to analyze dropout rates, demographic data, and baseline characteristics. The safety analysis set included all randomized subjects who received at least one dose of the drug and had at least one safety parameter recorded. This dataset was used for safety statistical analysis. The pharmacokinetics (PK) concentration analysis set consisted of all randomized subjects who received at least one dose of the study drug and had at least one plasma concentration data point. The PK parameter analysis set (PKPS) included all randomized subjects who received at least one dose of the study drug and had at least one valid PK parameter recorded during the trial. The bioequivalence analysis set (BES) included evaluable PK parameters from at least one period.

Statistical analyses were performed using SAS (v9.4, SAS Institute Inc., United States), and PK parameters were calculated with WinNonlin (v8.1, Certara, United States). Demographic characteristics (e.g., age, sex, and BMI) and baseline data were summarized using descriptive statistics. Continuous variables were compared using Student’s t-test, and categorical variables were analyzed using Fisher’s exact test. Following logarithmic transformation, the analysis of variance was applied to the primary PK parameters to estimate intergroup standard deviations. For bioequivalence evaluation, geometric mean ratios (GMRs; test/reference mean ratio) and 90% confidence intervals (CIs) were derived. The Wilcoxon signed-rank test was used to analyze the median and 90% CIs of T_max_ values.

## 3 Result

### 3.1 Subjects

A total of 54 subjects were screened for this trial. They provided written informed consent after being fully informed about the trial procedures and associated risks. A total of 54 subjects underwent screening, with 30 of them failing the screening and 24 meeting the eligibility criteria. All randomized subjects adhered to the protocol requirements during the randomization process, resulting in 12 subjects being allocated to each sequence group (R-T sequence group or T-R sequence group). All 24 subjects completed the trial and were included in the FAS to analyze demographic data and baseline characteristics. No statistically significant differences were observed in baseline characteristics between the two groups. Relevant demographic data are presented in Table 1.

**TABLE 1:** Demographic characteristics of 24 healthy subjects.

Variable	Fasted/fed state		
	Group (N)		Total
	T-R (12)	R-T (12)	24
Age, y, mean ± SD	36.3 ± 9.3	35.6 ± 9.6	36.1 ± 9.5
Female sex, no (%)	12 (100.00%)	12 (100.00%)	24 (100.00%)
Ethnic, Han, no (%)	12 (100.00%)	12 (100.00%)	24 (100.00%)
Height, cm, mean ± SD	159.6 ± 4.2	158.5 ± 5.4	159.1 ± 4.8
Weight, kg, mean ± SD	56.9 ± 5.9	56.9 ± 5.4	56.9 ± 5.5
BMI, kg/m^2^, mean ± SD	22.3 ± 1.6	22.7 ± 2.0	22.5 ± 1.8

Abbreviations: BMI: body mass index, weight (kg)/[height (m)] SD: standard deviation; T-R: test–reference group; R-T: reference–test group.

### 3.2 Method validation

The developed UPLC-MS method demonstrated a linear range of 0.05–5.00 ng/mL (r^2^ > 0.998) with a lower limit of quantification of 0.0500 ng/mL. Precision and accuracy were evaluated using quality control (QC) samples at four levels: low (0.150 ng/mL), geometric mean (0.750 ng/mL), medium (2.00 ng/mL), and high (3.75 ng/mL). Intra- and inter-day precision (expressed as % coefficient of variation) ranged from 3.7% to 4.6%, while accuracy (expressed as % relative error) was within ±2.0% for all QC levels.

To ensure reproducibility, the incurred sample reanalysis (ISR) was performed on 193 study samples. All ISR results met the acceptance criteria, with 100% of the reanalyzed samples showing a percent difference (%D) between −15.1% and +16.5%. This fulfilled the requirement that at least two-thirds of the reanalyzed samples must have a %D within ±20.0%.

### 3.3 PK parameters

**FIGURE 2:**
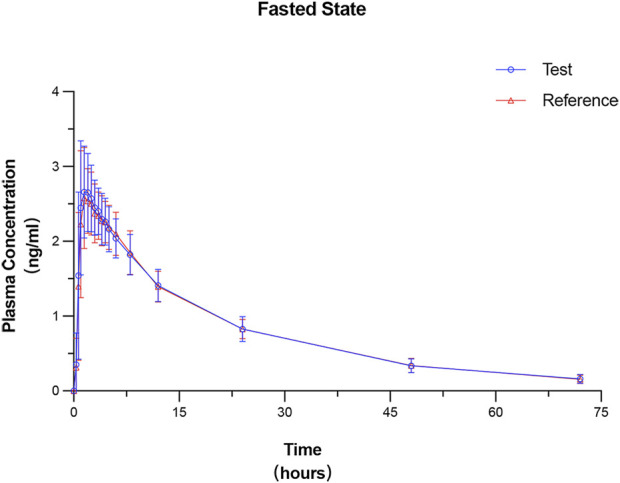
Mean plasma concentration–time profiles of prucalopride following a single 1-mg oral dose of the test and reference formulations under the fasted state. Data are presented as mean ± SD (error bars).

**FIGURE 3:**
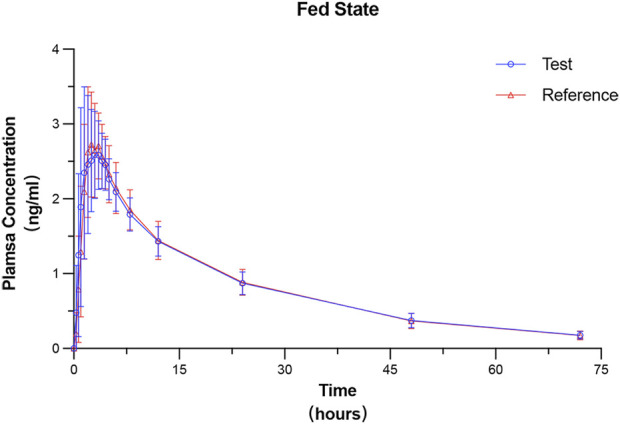
Mean plasma concentration–time profiles of prucalopride following a single 1-mg oral dose of the test and reference formulations under the fed state. Data are presented as mean ± SD (error bars).

All 24 subjects received at least one dose of the test formulation, had valid PK parameters, and were included in the PKPS for PK analysis. The plasma concentration–time curves under fasted and fed states were plotted on the basis of the actual blood sampling time points and are presented in Figures 2, 3, respectively. The PK parameters derived from non-compartmental analysis under the fasted and fed states are summarized in Table 2. In the fasted trial, no significant differences were observed between the test formulation and the reference formulation in key PK parameters, including C_max_ (3.00 ± 0.67 ng·mL^−1^ vs. 2.94 ± 0.61 ng·mL^−1^), t_1/2_ (19.26 ± 2.83 h vs. 19.56 ± 3.06 h), and AUC_0-t_ (56.55 ± 8.38 ng·h·mL^−1^ vs. 56.28 ± 7.70 ng·h·mL^−1^). Similar results were obtained in the fed trial.

**TABLE 2:** PK parameters of prucalopride in fasted and fed states.

PK parameters	Fasted state		Fed state	
	TF	RF	TF	RF
λ_z_ (h^−1^)	0.04 ± 0.01 (15.96%)	0.04 ± 0.00 (12.24%)	0.03 ± 0.00 (11.83%)	0.04 ± 0.00 (14.16%)
t_1/2_(h)	19.26 ± 2.83 (14.69%)	19.56 ± 3.06 (15.65%)	20.62 ± 2.54 (12.30%)	20.01 ± 2.76 (13.80%)
T_max_ (h)	1.75 (0.67,5.00)	1.50 (0.67,6.02)	2.75 (0.67,4.50)	2.25 (1.00,4.50)
C_max_ (ng⋅mL^−1^)	3.00 ± 0.67 (22.30%)	2.94 ± 0.61 (20.83%)	3.05 ± 0.72 (23.67%)	3.10 ± 0.49 (15.71%)
AUC_0-t_ (ng⋅h⋅mL^−1^)	56.55 ± 8.38 (14.82%)	56.28 ± 7.70 (13.68%)	58.50 ± 8.38 (14.32%)	58.71 ± 9.29 (15.82%)
AUC_0∼∞_(ng⋅h⋅mL^−1^)	61.18 ± 10.27 (16.78%)	60.90 ± 9.73 (15.99%)	63.94 ± 9.93 (15.54%)	63.93 ± 11.15 (17.45%)

Note: Tmax is presented as the median (minimum–maximum). Other data are presented as geometric mean ± SD (CV%).

Abbreviations: TF: test formulation; RF: reference formulation; CV: coefficient of variation; λ_z_: terminal elimination rate constant; t_1/2_: terminal elimination half-life; T_max_: time to maximum concentration; C_max_: maximum concentration; AUC_0-t_: area under the concentration to time curve from time zero to time t; AUC_0∼∞_: area under the concentration to time curve from time zero to infinity.

The test formulation exhibited a prolonged T_max_ under fed conditions compared to the fasted state (2.75 h vs. 1.75 h). A similar delay in T_max_ was observed for the reference formulation (2.25 h vs. 1.50 h). Nonparametric analysis (Wilcoxon signed-rank test) results of T_max_ are summarized in Table 3, confirming that T_max_ was increased under the fed state. However, no statistically significant differences in T_max_ were observed between the test and reference formulations within the same trial condition.

**TABLE 3:** Wilcoxon signed-rank test results of T_max_.

Parameter	Fasted state		Fed state	
	TF	RF	TF	RF
Mean ± SD (h)	1.87 ± 1.00	2.11 ± 1.35	2.67 ± 1.34	2.63 ± 0.97
95% CI (h)	1.45,2.29	1.54,2.68	2.11,3.24	2.21,3.05
Statistic	−19.5		−1.0	
P value	0.319		0.973	

Note: Significance level set at α = 0.05.

Abbreviations: TF: test formulation; RF: reference formulation; CI: confidence interval; SD: standard deviation.

### 3.4 Bioequivalence analysis

In the fasted-state trial, all subjects’ datasets were included in the BES for bioequivalence analysis. The GMRs [T/R (90% CI)] for C_max_, AUC_0-t_, and AUC_0-∞_ in the fasted-state trial were 101.59% (96.93–106.48), 100.30% (97.72–102.95), and 100.27% (97.46–103.15), respectively. In the fed-state trial, one subject vomited at 1 h post-dose (within twice the median T_max_), leading to their exclusion from the BES due to the potential impact on absorption. The GMRs (90% CI) of the fed state were C_max_: 98.70% (91.84%−106.08%), AUC_0-t_: 101.42% (98.71%–104.21%), and AUC_0-∞_: 101.83% (99.03%–104.71%). The 90% CIs for all parameters fell within the range of 80%–125%, which aligns with the standard bioequivalence acceptance criteria specified in the NMPA Guideline. The outcome of the bioequivalence assessment is shown in Table 4.

**TABLE 4:** Bioequivalence evaluation of the generic and reference formulations under the fasted and fed states.

Parameter	Fasted state (N = 24)			Fed state (N = 23)		
	T/R ratio (%)	90% CI	CV (%)	T/R ratio (%)	90% CI	CV (%)
LN(C_max_)	101.59	96.93–106.48	9.50%	98.70	91.84–106.08	14.37%
LN (AUC_0-t_)	100.30	97.72–102.95	5.26%	101.42	98.71–104.21	5.34%
LN (AUC_0-∞_)	100.27	97.46–103.15	5.73%	101.83	99.03–104.71	5.50%

Note: Bioequivalence was concluded if 90% CI for the geometric mean ratio of pharmacokinetic parameters fell entirely within the acceptance range of 80.00%–125.00%.

Abbreviations: T/R: test-to-reference geometric mean ratio; CI: confidence interval; CV: coefficient of variation; LN: natural log-transformed.

### 3.5 Clinical safety

AEs observed during the study are summarized in Table 5. In the fasted-state trial, diarrhea was the most common AE, occurring in 70.8% (17/24) of the subjects for both the test and reference formulations. Hypotension was reported in 12.5% (3/24) of test formulation recipients compared to 8.3% (2/24) for the reference group. Headache and tachycardia were observed exclusively in the reference group (8.3%, 2/24 each). No cases of vomiting or elevated low-density lipoprotein cholesterol (LDL-C) were observed in the fasted state.

**TABLE 5:** Summary of AEs under the fasted and fed states.

Adverse event	Fasted state				Fed state			
	TF (n = 24)		RF (n = 24)		TF (n = 24)		RF (n = 24)	
	Subjects (n, %)	Case (n)	Subjects (n, %)	Case (n)	Subjects (n, %)	Case (n)	Subjects (n, %)	Case (n)
Diarrhea	17 (70.8%)	17	17 (70.8%)	17	17 (70.8%)	17	13 (54.2%)	13
Vomiting	0	0	0	0	0	0	1 (4.2%)	1
Hypotension	3 (12.5%)	3	2 (8.3%)	2	3 (12.5%)	3	0	0
Headache	2 (8.3%)	2	2 (8.3%)	2	0	0	0	0
Tachycardia	0	0	2 (8.3%)	2	0	0	0	0
High LDL-C	0	0	0	0	3 (12.5%)	3	0	0

Abbreviations: TF: test formulation; RF: reference formulation.

In the fed trial, diarrhea incidence for the reference formulation was slightly lower (54.2%, 13/24) than it was for the test group (70.8%, 17/24). Vomiting occurred in 4.2% (1/24) of reference formulation recipients, while elevated LDL-C (12.5%, 3/24) and transient hypotension (12.5%, 3/24) were observed in the test group. All lipid abnormalities (e.g., elevated LDL-C and triglycerides) resolved spontaneously without intervention. Additionally, no other severe AEs were observed during the study.

## 4 Discussion

This randomized, two-period, crossover bioequivalence trial evaluated the PK, safety, and food effect of a generic prucalopride formulation compared to the reference formulation (Resolor^®^) in healthy Chinese female subjects. A total of 24 participants were enrolled, and all completed both the fasted and fed states of the study without protocol deviations or discontinuations.

The critical PK parameters (C_max_, AUC_0-t_, and AUC_0-∞_) of the two formulations met the bioequivalence criteria in both the states, with no statistically significant differences in T_max_. Additionally, the fed state was associated with prolonged T_max_ values, suggesting altered absorption kinetics. The delayed T_max_ in the fed state was likely associated with the delayed gastric emptying induced by high-fat meals and the high lipophilicity of prucalopride (Jin et al., 2023). These findings validate the clinical equivalence of the generic formulation across dietary conditions and support optimized dosing strategies such as postprandial administration to mitigate gastrointestinal irritation. Furthermore, the identical PK profiles of the generic and reference formulations under both fasted and fed states confirm equivalence in excipient composition and manufacturing processes (e.g., disintegration time and dissolution rate).

The comparison of PK parameters between the generic formulation and reference formulation, as reported in the literature, revealed a higher AUC value in Caucasian individuals (Smith et al., 2012; Flach et al., 2016). This difference may be attributed to ethnic variations in gastrointestinal absorption or population-specific factors, such as age-related declines in renal function that affect drug clearance mechanisms. Additionally, no significant differences in T_max_ or t_1/2_ were observed among different ethnic groups or sexes (Camilleri et al., 2008). Prior studies on prucalopride in Chinese cohorts reported similar C_max_ and T_max_ values (Zhou et al., 2020; Chen et al., 2012). By contrast, this trial showed a lower AUC value. This discrepancy could be attributed to sex-related differences as variations in the sex composition within the study cohort may influence drug distribution and elimination processes (Guidi et al., 2022).

In terms of safety, the AEs reported in previous studies were similar to that observed in this study (Diederen et al., 2015; Frampton, 2009). There was no difference in the incidence of AEs between the fed and fasted states. No serious AEs occurred, and the test formulation demonstrated good tolerability. Although 34 cases of diarrhea in 20 subjects in the fasted trial and 30 cases of diarrhea in 21 subjects in the fed trial were observed, the AEs were transient and within an acceptable range, consistent with the pharmacological action of prucalopride. Other AEs, such as headache and hypotension, may be associated with the activation of the 5-HT4 receptor. Additionally, some laboratory abnormalities, such as elevated LDL-C, were observed but resolved during follow-up.

Previous reports have indicated that nonselective 5-HT4 receptor agonists can lead to QT interval prolongation or other cardiovascular AEs (De et al., 2008). However, prucalopride, being a highly selective 5-HT4 receptor agonist, primarily targets the 5-HT4 receptors in the colonic myenteric plexus and has minimal affinity for the cardiac 5-HT4 receptor (Jadallah et al., 2014). In this trial, no electrocardiographic abnormalities (e.g., QT interval prolongation) or cardiotoxicity-related events were observed. Although two mild cases of tachycardia (8.3%) were reported, these events were transient and resolved rapidly without clinical consequences. However, due to the limited sample size of this trial (n = 24), all potential pharmacodynamic and safety profiles of the drug, particularly cardiovascular risks associated with long-term use, may not be comprehensively assessed. Therefore, longer-term clinical studies are still required to further validate the cardiac safety of this generic formulation, especially in elderly patients or high-risk populations with comorbid cardiovascular diseases.

## 5 Conclusion

This randomized crossover study confirmed the bioequivalence of a generic prucalopride formulation to the reference product (Resolor^®^) in healthy Chinese women under both fasted and fed states. Both formulations demonstrated comparable safety profiles and were well tolerated.

## Data Availability

The original contributions presented in the study are included in the article/supplementary material, and further inquiries can be directed to the corresponding authors.
